# Natural Selection and Neutral Evolution Jointly Drive Population Divergence between Alpine and Lowland Ecotypes of the Allopolyploid Plant *Anemone multifida* (Ranunculaceae)

**DOI:** 10.1371/journal.pone.0068889

**Published:** 2013-07-18

**Authors:** Jamie R. McEwen, Jana C. Vamosi, Sean M. Rogers

**Affiliations:** 1 University of British Columbia, Department of Botany, Vancouver, BC, Canada; 2 University of Calgary, Department of Biological Sciences, Calgary, Alberta, Canada; The University of Queensland, St. Lucia, Australia

## Abstract

Population differentiation can be driven in large part by natural selection, but selectively neutral evolution can play a prominent role in shaping patters of population divergence. The decomposition of the evolutionary history of populations into the relative effects of natural selection and selectively neutral evolution enables an understanding of the causes of population divergence and adaptation. In this study, we examined heterogeneous genomic divergence between alpine and lowland ecotypes of the allopolyploid plant, *Anemone multifida*. Using peak height and dominant AFLP data, we quantified population differentiation at non-outlier (neutral) and outlier loci to determine the potential contribution of natural selection and selectively neutral evolution to population divergence. We found 13 candidate loci, corresponding to 2.7% of loci, with signatures of divergent natural selection between alpine and lowland populations and between alpine populations (Fst  = 0.074–0.445 at outlier loci), but neutral population differentiation was also evident between alpine populations (F_ST_  = 0.041–0.095 at neutral loci). By examining population structure at both neutral and outlier loci, we determined that the combined effects of selection and neutral evolution are associated with the divergence of alpine populations, which may be linked to extreme abiotic conditions and isolation between alpine sites. The presence of outlier levels of genetic variation in structured populations underscores the importance of separately analyzing neutral and outlier loci to infer the relative role of divergent natural selection and neutral evolution in population divergence.

## Introduction

Population divergence and speciation have been driven in large part by the joint effects of neutral evolution and natural selection [1], [2]. Variation in environmental conditions between populations, ecological opportunity during colonization or modification of a habitat can drive differentiation between populations or ecotypes [3]–[5]. Reductions in gene flow between populations due to divergent selection can also enhance the impact of selectively neutral mechanisms of evolution (e.g. genetic drift), further accelerating differentiation between populations [6]–[8]. These isolating effects of adaptation can eventually lead to widespread genomic differentiation between populations or ecotypes, eventually leading to speciation [9]–[12]. Isolation by adaptation has been observed in a number of cases by the genetic breakdown of individuals hybridized between reproductively isolated ecotypes [13]–[16], although processes such as polyploidy may still bridge the gap between species at the later stages of speciation [17].

While natural selection is often the cause of speciation and ecological divergence [2], [18], selectively neutral evolution is also a prominent mode of speciation and population divergence [19], [20]. Genetic drift can lead to the fixation or loss of alleles [21], while mutations are an important source of genetic diversity [22]. Demographic events, such as population bottlenecks or the founder effect, can accelerate allele fixation in a population [23], [24], while gene flow can potentially erode population differentiation and lessen the impacts of selection or drift [25]. While neutral evolution and natural selection are not mutually exclusive in their effects on the evolution of genes and populations, accounting for selectively neutral evolution is necessary for an accurate determination of the causes of genetic variation and population structure [26]–[28]. For example, the presence of population structure suggests evolutionary divergence has progressed to the point that neutral evolution will be a major determinant of genetic variation, increasing the likelihood that loci with putative signatures of natural selection have evolved by selectively neutral mechanisms [29].

The primary focus of most population genomic investigations is on detection and patterns of variation of outlier loci [30], [31], while the potential role of selectively neutral evolution in population divergence is often not directly addressed (with neutral markers being primarily used to determine population structure). However, the identification of candidate loci that show signatures of natural selection implicitly identifies non-outlier loci that should be selectively neutral between populations. The incorporation of information about population structure due to selectively neutral evolution is an important step in addressing alternate explanations for population differentiation at outlier loci, which are more likely in structured populations [32].

In this study, we conduct a genome scan for putative signatures of natural selection between alpine and lowland ecotypes of the allopolyploid plant, *Anemone multifida*. Alpine and lowland habitats differ extensively in abiotic and biotic conditions [33], and in numerous cases have been associated with divergent adaptation [34]–[40]. The generally extreme nature of alpine environments has in some cases selected for alpine specialization, and speciation itself may be accelerated in alpine habitats [33], [41]–[44]. The ecological differences between lowland and alpine environments can affect patterns of dispersal, rates of population divergence, and speciation [41], [45]–[47]. The isolation of mountain tops amongst intervening temperate environments can reinforce this differentiation through restricted gene flow [45]–[49]. In contrast, the potentially more homogeneous abiotic environment across lowland habitats and the lack of major barriers to dispersal may allow more extensive gene flow between lowland populations [50].

The goals of this study were to determine 1) whether the genome of *A. multifida* includes outlier loci that may have been the target of natural selection, 2) whether populations in the same environment possess similar alleles that would indicate that natural selection (vs. genetic drift) has led to alpine and lowland adaptation, and 3) if selectively neutral evolution has driven population divergence. To accomplish these goals, we conducted a genome scan for outlier loci amongst multiple alpine and lowland populations. Incorporating both binary and peak height AFLP data [51], we split the resulting genetic data into outlier and neutral locus datasets to separately analyze patterns of population differentiation caused by natural selection and selectively neutral evolution. This method delineates the effect of neutral evolution in determining population divergence, improving the accuracy of inference about the cause of outlier levels of genetic differentiation amongst the genome of divergent ecotypes.

If natural selection has affected the genome of *A. multifida*, outlier loci should show limited differentiation between populations in the case of balancing selection, or extensive differentiation if divergent selection has had an effect. Alternatively, if natural selection has not affected specific sites throughout the genome, there should be no evidence of outlying loci. Analysis across multiple populations from each environment allows separation of demographic and environmental factors in determining genetic population structure, and identifies whether populations diverged primarily due to selectively neutral evolution. If so, neutral evolutionary processes, such as gene flow or genetic drift, have affected population evolution, in which case populations should be differentiated at the neutral loci. In contrast, high gene flow and/or negligible genetic drift should generate limited genomic differentiation between populations.

## Materials and Methods

### Study System

*Anemone multifida* is a widespread species that occupies habitats from sea level to high alpine. *A. multifida* is hypothesized to be an allotetraploid based on observations of two distinct chromosome sets, one of which is similar to chromosomes from a clade of alpine specialist species, whereas the other set is more similar to chromosomes from a lowland clade [52], [53]. Therefore, *A. multifida* may possess alternate copies of alleles that are advantageous in alpine environments (from the “alpine” chromosome set) and lowland environments (from the “lowland” chromosome set), which may explain its wide habitat range [52], [53]. Both sympatric and allopatric populations of *A. multifida* exhibit extensive morphological variation [52], [53]. Throughout its North American distribution there are white, red and pink flowers, whereas only white flowers occur in South America.

### Sampling

**Figure 1:**
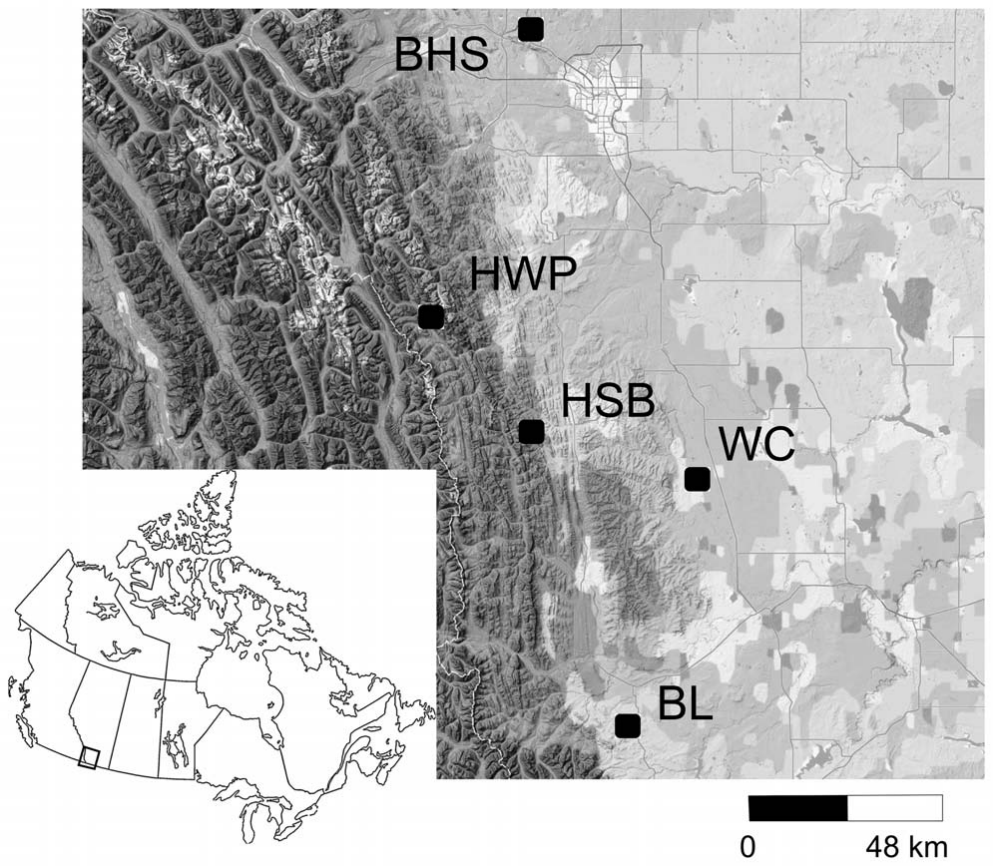
Location of populations sampled from Alberta, Canada, during June and July, 2011. Populations BHS, WC and BL are lowland and HWP and HSB are alpine sites.

Leaf tissue was sampled from approximately 30 *A. multifida* individuals during flowering from each of two alpine and three lowland sites Alberta, Canada, during June and July, 2011 (Table 1; Figure 1), with the permission of Alberta Tourism Parks and Recreation. Within populations, plants were sampled along a transect with a minimum distance of 7 m between individuals. Leaf material was placed in plastic bags with silica gel for storage. Floral colour and plant height were measured in the field as these are variable traits in this species. To measure floral colour, petals were collected from individuals displaying all floral colour morphs (white, red and pink) in a sample from Big Hill Springs, Alberta, placed in a cooler to prevent pigment degradation during transport, and scanned the same day with an Ocean Optics USB 2000 spectrophotometer to assess floral colour [54]. Floral colours generally fell into white (uniform transmittance across the visual spectrum), red (transmittance in visual-red wavelengths), and pink (slightly higher uniform transmittance and lower transmittance in visual-red spectrum) with no UV reflectance, so remaining floral colour phenotypes were scored according to their visual colour without a spectrophotometer. Above ground plant height was measured on live individuals from the base of the plant at the soil to the tallest flowering shoot using a tape measure.

**Table 1:** Location and elevation of sites from which *A. multifida* was sampled.

Population	Final Sample Size	Latitude (°N)	Longitude (°W)	Elevation (m)
Big Hill Springs	24	51.251	114.386	1229
Beauvais Lake	25	49.415	114.092	1472
Willow Creek	21	50.117	113.777	1055
Hailstone Butte	29	50.205	114.445	2080
Highwood Pass	24	50.604	114.984	2377

The lowland populations were from Big Hill Springs (BHS), Beauvais Lake (BL), and Willow Creek (WC), while alpine populations were from Hailstone Butte (HSB) and Highwood Pass (HWP).

The environmental differences between populations were not quantified in this study, but are available from other sources for populations adjacent to the sites used in this study [34]. Alpine environments are generally characterized as having extreme abiotic conditions, having higher levels of photosynthetically active radiation (PAR), stronger winds, lower temperatures and shorter growing seasons than lowland habitats (Table 2; [34]). In lowland environments, the potential effects of more intense competition are evident in the lower soil moisture and nutrient content, as well as the greater biomass and height at herbaceous plant layers (Table 2; [34]).

**Table 2:** The environmental differences between an alpine and a lowland environment near the sites sampled in this study, based on [34].

	Alpine	Lowland
Elevation (m)	2453	1310
PAR (µgE/sm^2^)	2242	1627
Wind (m/s)	6.6	2.8
Growing season temperature (°C)	7.9	14.7
Herb layer biomass (g/m^2^)	142.3	572.2
Herb layer height (cm)	15.3	72.9
Soil moisture (% wt)	60.9	35.5
Soil NH_3_ (ug/g dry mass)	62.1	13.9

Only soil NH_3_ is provided, as other soil nutrients (NO_3_ and PO_4_) follow a similar pattern (higher nutrient and organic content in alpine than lowland). PAR - photosynthetically active radiation.

### DNA Isolation and AFLP

DNA was extracted from silica-dried leaf tissues using a standard CTAB/chloroform DNA extraction protocol [55]. DNA quality was determined with agarose gel electrophoresis to assess any DNA degradation, and a Beckman Coulter DTX 880 Multimode Detector spectrophotometer (Beckman Coulter, Brea, CA, USA) was used to assess contamination from protein and RNA and quantify DNA. DNA concentration was standardized to 150 ng/µL and a total of 750 ng was used for amplified fragment length polymorphism (AFLP) analysis following the Amplification Kit for Regular Plant Genomes (Applied Biosystems, Carlsbad, CA, USA) using the restriction enzymes EcoR I and Mse I (New England BioLabs, Ipswich, MA, USA). DNA was digested by incubating overnight with the restriction enzymes, T4 DNA ligase, NaCl, BSA and the complementary adaptors, checked for complete digestion on an agarose gel and diluted to a 10X concentration in water for preselective amplification. Preselective amplification was conducted with the supplied reagents according to the manufacturer’s instructions, and checked to verify that amplification occurred in the 100–1500 bp range on an agarose gel. Preselective amplification was done with primers non-selective primer that were complementary to the ligated adaptors. Preselective product was diluted to a 5X concentration for selective amplification. Selective amplification was performed on the preselective product with three MseI - EcoRI adaptors CAA-ACG, CAC-ACG, and CTC-AGG with the AFLP Amplification Core Mix PCR master mix (Applied Biosystems, Carlsbad, CA, USA). AFLP fragments were separated with simplex reactions on an Applied Biosystems 3500xL Genetic Analyzer (Applied Biosystems, Carlsbad, CA, USA).

Allele sizes (in base pairs) were determined by reference to the internal sizing standard (GS-500 LIZ) in the software GENEMAPPER v4.0 (Applied Biosystems, Carlsbad, CA, USA). Fragments between 100–500 bp were scored using automatic allele binning in Genemapper, with a cut-off intensity of 100 fluorescent units to minimize false-allele calling. Polymorphic peaks identified in Genemapper were then manually checked for quality and consistent scoring. AFLP alleles with multiple peaks were discarded due to the unreliable sizing of the fragments. AFLP alleles with amplification at or near the 100 fluorescent unit cut off were manually checked for consistent scoring, as peaks with amplification just below the threshold can be a source of allele drop out [56]. The identified alleles were first checked against five DNA sample replicates on different gels. Loci that were inconsistently scored between DNA replicates were used to quantify the error rate and then removed from the final data set. Samples that had weak amplification or high noise across the electropherogram were also discarded to reduce allele dropout and false-allele calling stemming from failed or non-optimal PCR conditions, leaving 479 loci in the final dataset.

### Detection of Outlier Loci

Genemapper provides the option of exporting both the dominant (binary, present or absent allele information) and peak height data from each allele (if the allele is present). Most analyses have chosen to use the dominant data as the basis of genotyping individuals, but we also incorporated peak height data for comparison [51]. We used BayeScan [37], [51], [57], which natively accepts both data types, to identify outlier AFLP loci with both dominant and peak height data. Peak height data were standardized by subtracting individual mean peak height from amplification intensity at each locus. There was no apparent codominant grouping of alleles, which is consistent with previous attempts at using AFLP peak height as codominant markers [51], [58]. The method used by BayeScan instead uses the distribution of peak height data within populations to directly estimate F-statistics [51]. In this study, we considered a log posterior odds >10 as indicating that a particular locus is an outlier, as in previous investigations [37], [51], [57], [59], [60]. We used a burn-in of 50,000 iterations, and a sample size of 10,000 with a thinning interval of 50 (following [37], [57]). The number and identity of loci determined to be outliers with peak height and dominant data were compared to determine if any major discrepancies occurred when using either form of AFLP data. Further analyses were used only on binary data as peak height data is not compatible with the majority of software available for population genetics.

### Genetic and Population Structure Analyses

The number of genetic clusters was first identified with a principal components analysis of AFLP data using R statistical software [61], and any apparent clustering in the neutral and outlier loci data along the principal component axes was assessed [62]. PCA can be useful for visualization but alone does not constitute a rigorous test of genetic clustering [63]. Therefore, we also used the individual assignment-based approach implemented in STRUCTURE version 2.3.3 to determine the number of distinct genetic groups (K) in the data [63]–[66]. We used the admixture model for this study, as it seemed most reasonable considering the ecological and evolutionary history of *A. multifida*. STRUCTURE analyses assume diploid inheritance, which is potentially violated for many of the AFLP loci in this study of an allopolyploid species. Simulations using a burn-in of 10000 iterations and 10000 MCMC replicates after burn-in were used to determine the probability of the model assuming 1 to 7 populations. These simulations were replicated 10 times at each level of K, allowing for a correction of the STRUCTURE results following [67].

We analyzed the distribution of genetic variation among and within populations with an analysis of molecular variance (AMOVA) analysis using GenAlEx [68]–[70]. AMOVA can be used to test for genetic variance among populations (i.e. significant population structure), and differentiation amongst individuals within populations (i.e. the population reproduces sexually). The significance of the proportion of variance attributed to among-population effects (φ) is tested by comparing the observed φ to a distribution of φ based on simulated populations of randomly assigned individuals [68].

To estimate and test genetic population structure between sampled sites (F_ST_), we used AFLPsurv v1.0 [71], which assesses F_ST_ from the frequency of the null AFLP allele using a number of options. We chose a Bayesian method with non-uniform prior distribution of allele frequencies assuming Hardy-Weinberg conditions were met [72], which has been regularly used for estimating null allele frequencies in AFLP studies [71], [73]. AFLPsurv assumes that individuals are diploid, potentially leading to lower estimates of population differentiation in polyploid species (i.e. there may be higher heterozygosity within populations due to the possibility of more than two alleles at each locus).

Determination of patterns of population structure at the neutral and outlier loci assumes that loci are transmitted independently of each other. Instead, loci may be in gametic-phase disequilibrium, tending to vary together within and between populations, because of physical linkage or other non-random associations between alleles. In such cases, regions of the genome under selection typically impact adjacent genomic regions [11], [74]. Testing for gametic-phase disequilibrium amongst outliers is necessary to isolate the effects of genetic hitchhiking from drift or selection at each locus. To test for gametic-phase disequilibrium amongst outlier loci, we used MultiLocus 1.3 [75], which calculates the index of association [76]–[78] by comparing the number of loci that are different in pairwise comparisons of all individuals.

### Phenotype Analyses

Population differences between floral colour and plant height data were assessed to determine whether environment or demography may have affected the evolution of these phenotypes. All statistical tests were done in R statistical software [61]. Differences in the frequencies of floral colour morphs among populations were assessed with a chi-square test of independence. ANOVA was used to test for differences in plant height among populations. To determine whether population differences in floral colour distribution or mean plant height represented plasticity or had a genetic basis, we tested whether there were genetic associations with the measured phenotypes. Multiple Spearman correlations were used to detect associations between phenotypes and each allele, controlling for multiple comparisons with false discovery rate using the “fdr” option in R [79].

## Results

### AFLP and the Detection of Outlier Loci

**Figure 2:**
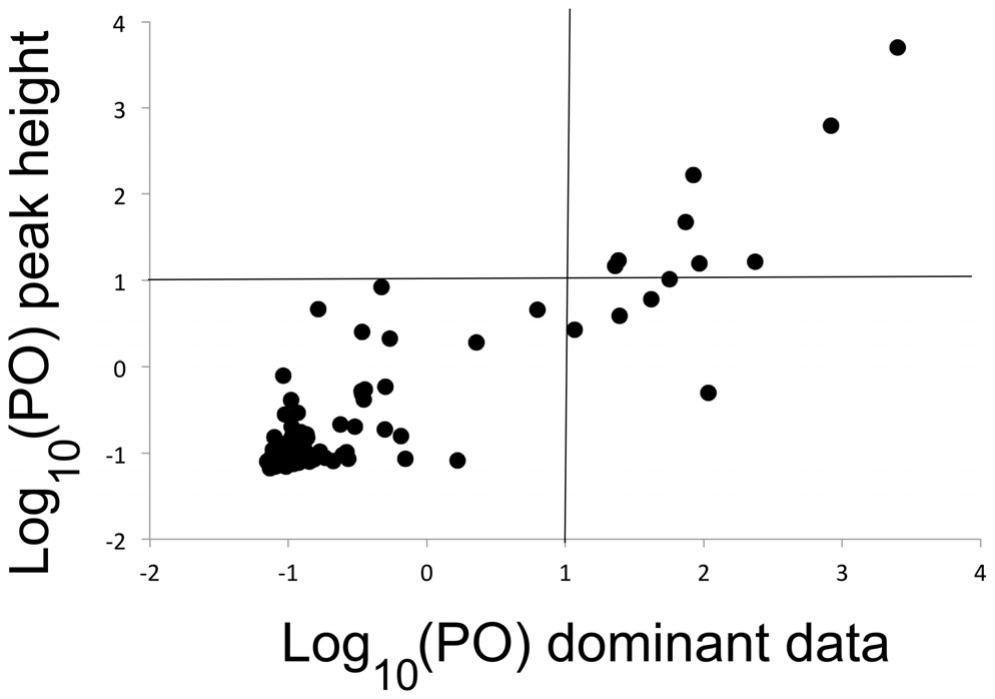
Comparison of the relative log_10_(PO) scores from BayeScan for each AFLP locus using the peak height and dominant AFLP data. The log posterior odds scores (PO) measures the probability a particular locus is an outlier when compared to the probability it is not an outlier (a higher score would indicate a higher probability of being an outlier). A log PO threshold of 1 is commonly used to delineate outlier from non-outlier loci [37].

A total of 759 markers amplified, of which 511 remained after discarding monomorphic markers and those with poor peak quality. There were 32 loci that were incorrectly scored between DNA replicates (approximately 6.26%), leaving 479 markers in the final dataset (Table 3). Amongst all alpine and lowland populations 13 loci (2.7%) were significant outliers amongst the dominant AFLP data, and nine were outliers (1.9%) based on peak height (all of which were also identified with the binary data). Overall, loci assessed with peak height had lower log posterior odds than the dominant data (Figure 2). The false-discovery rate for the dominant data was 0.022, almost half that for peak height (0.041). The power (1– false negative rate) for peak height was 0.893, which was lower, but in a similar range, to the power for the dominant data at 0.898. All outlier loci had a positive α, suggesting divergent selection [57].

**Table 3:** AFLP primer pair characteristics, including N_BANDS,_ the number of bands scored, N_SAMPLES_, the number of samples successfully scored, H_E_, expected heterozygosity, H_E_^primer^, expected heterozygosity averaged over primer combinations, H_E_^pop^, the expected heterozygosity averaged over populations, P, the proportion of polymorphic markers, and P_mean,_ the mean proportion of polymorphic markers.

		EcoRI-CAA MseI-ACG	EcoRI-CAC MseI-ACG	EcoRI-CTC MseI-AGG	H_E_^primer^
	Dye	JOE	JOE	JOE	
	N_BANDS_	133	163	183	
	N_SAMPLES_	122	122	122	
BHS	H_E_	0.094	0.134	0.123	0.117
	P	0.233	0.307	0.295	
BL	H_E_	0.084	0.104	0.113	0.100
	P	0.248	0.294	0.295	
WC	H_E_	0.097	0.130	0.129	0.118
	P	0.308	0.368	0.355	
HSB	H_E_	0.104	0.127	0.131	0.121
	P	0.263	0.307	0.328	
HWP	H_E_	0.106	0.128	0.135	0.123
	P	0.301	0.344	0.388	
	H_E_^pop^	0.097	0.124	0.126	
	P_mean_	0.265	0.324	0.332	

### Population Structure of Neutral Loci

**Figure 3:**
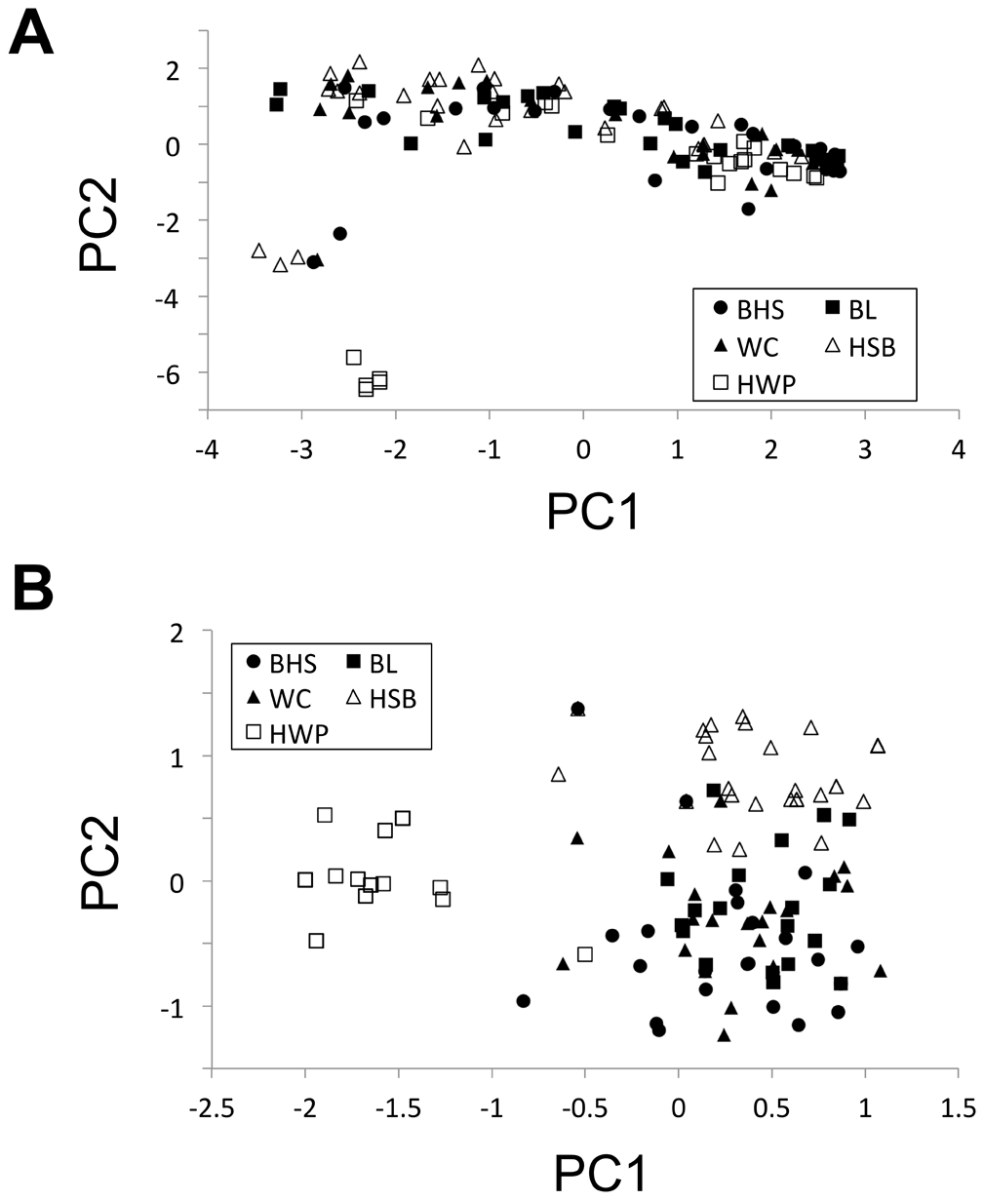
Scatterplot of the first two principal components of variation in AFLP genotype for non-outlier loci (A) and outlier loci (B). Alpine populations, HWP and HSB in open data points; lowland populations, BHS, BL, and WC in closed data points.

The first and second principal components of the neutral locus dataset explained approximately 13.0% and 10.3% of the overall variance, respectively. Additional principal components explained less than 6% of the variance. At the neutral loci, most individuals clustered together, regardless of population of origin, although there was variation along the PC1 axis, and a number of individuals deviated from the major cluster (Figure 3). Five individuals from the HWP alpine population and six individuals from mixed lowland and alpine populations clustered separately and deviated from the majority of individuals (Figure 3).

**Figure 4:**
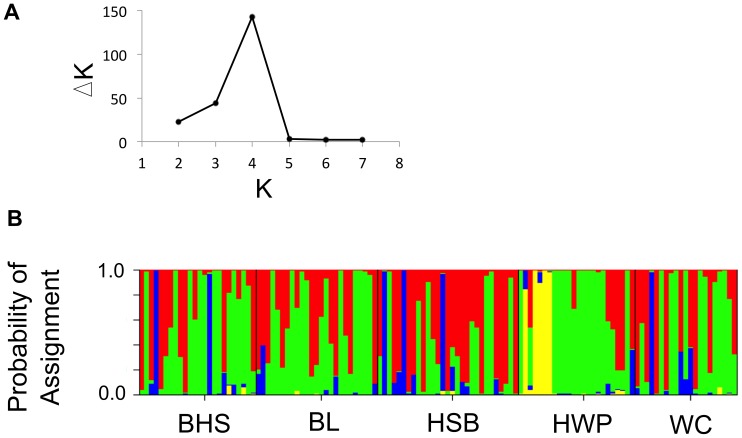
STRUCTURE results for neutral loci. A) The most likely K number of distinct genetic clusters at the neutral loci (top; denoted with the highest ΔK) following the correction method for STRUCTURE results by Evanno et al. (2005). B) Barplot showing the probabilities of individual assignment to each genetic cluster as assigned using neutral loci and assuming 4 genetic clusters. BHS, BL and WC are lowland and HSB and HWP are alpine sites.

STRUCTURE analysis suggested a most likely K of 4 or 5 distinct populations. After correction for the variance in probability estimates, the model of K  = 4 populations had the highest support, whereas K  = 5 had lower support than other models (Figure 4). The number of distinct genetic clusters within the data was therefore deemed to be 4 for future plotting and analyses. STRUCTURE identified unique groups in the HWP population (blue and yellow clusters in Figure 4), corresponding to the HWP and the HSB/BL/BHS groups as was found in the PCA. There were two additional major clusters (green and red, Figure 4), with a few individuals assigned roughly equally to both the red and green clusters overall (Figure 4).

AFLPsurv identified significant population structure amongst sampled sites at the neutral loci, with a global F_ST_ of 0.041 and a 99% upper limit F_ST_ of 0.021 (i.e. p<0.01). The HWP alpine population was significantly subdivided from all populations (Table 4). The lowland sites did not exhibit significant genetic structure, with F_ST_ values for all comparisons not significantly different from zero (Table 4). Approximately 9% of the molecular variation at neutral loci occurred between sites (AMOVA, *φ*_4,121_ = 0.089, p<0.001).

**Table 4:** F_ST_ estimates based on dominant data for all neutral (top panel) and outlier (bottom panel) loci for all pairs of populations.

	BHS	BL	HSB	HWP
BL	0.018			
HSB	0.019	**0.041**		
HWP	**0.072**	**0.095**	**0.067**	
WC	0.004	0.013	0.009	**0.057**
	**BHS**	**BL**	**HSB**	**HWP**
BL	0.074			
HSB	0.206	0.167		
HWP	0.338	0.429	0.445	
WC	0.092	0.032	0.118	0.365

Estimates that differed significantly from zero at p<0.01 are bolded, except in the outlier table in which all F_ST_ estimates were significantly greater than zero. The lowland populations are BHS, BL, and WC, and alpine sites are HSB and HWP.

### Population Structure of the Outlier Loci

**Figure 5:**
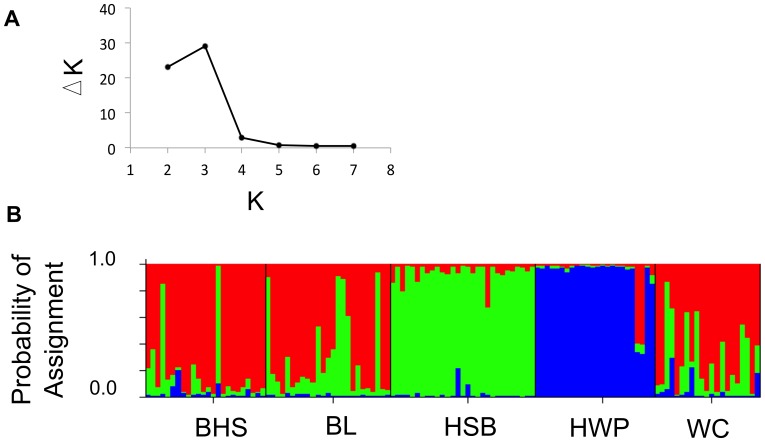
STRUCTURE results for the outlier loci. A) The most likely number of distinct genetic clusters, K, at the outlier loci (top; denoted with the highest ΔK) detected by STRUCTURE following the correction method of Evanno et al. (2005). B) Barplot showing the probability of individual assignment to each genetic cluster (bottom; represented by different colours) for outlier loci. Sites represented are lowland (BHS, BL, and WC) as well as alpine (HSB and HWP).

Using the 13 outlier loci identified in with the dominant AFLP data type, the first and second principal components of the outlier locus dataset explained approximately 29.5% and 16.8% of the overall variance, respectively. Further principal components explained less than 9% of the variance individually. Most individuals from the lowland sites grouped together (Figure 3). Alpine sites tended to cluster separately from each other and the lowland cluster, with particular divergence amongst the HWP-alpine group (Figure 3). STRUCTURE identified 3 or 4 distinct genetic clusters, with 3 clusters receiving highest support after variance correction (Figure 5). Three genetic clusters distinguished each alpine site and a lowland group, and a number of lowland individuals had high probabilities of assignment (80% or higher) to the HSB-alpine cluster (Figure 5).

All analyses of the outlier loci consistently identified three groups of individuals, corresponding to each alpine site and a lowland group. AFLPsurv identified significant population structure amongst sampled sites at the outlier loci, with a global F_ST_ of 0.255 and a 99% upper limit F_ST_ of 0.022 (i.e. p<0.01). F_ST_ differed significantly from zero between all population pairs, but was highest between lowland and alpine environments as well as alpine sites (Table 4). Approximately 15% of the genetic variance occurred between environments in the outlier loci (AMOVA, *φ*_1,121_ = 0.152, p<0.01).

The frequencies of alleles appeared to vary the most between alpine and lowland populations (Table 5), but there were too few populations sampled to assess the significance of any differences at each locus. Only one locus had similar allele frequencies between the two alpine populations (locus 58); otherwise allele frequencies differed most between alpine sites. The mean difference in allele frequencies was 0.179 between HSB-alpine and the lowland, 0.393 between lowland and HWP-alpine, and 0.461 between alpine sites (Table 5). No genetic associations were detected amongst pairs of outlier loci.

**Table 5:** Allele frequencies of outlier loci each lowland (BHS, BL and WC) and alpine (HSB and HWP) site.

Outlier	BHS (lowland)	BL (lowland)	WC (lowland)	HSB (alpine)	HWP (alpine)
locus1	0.156	0.217	0.158	0.691	0.221
locus58	0.479	0.211	0.333	0.030	0.013
locus78	0.015	0.009	0.043	0.010	0.715
locus169	0.858	0.924	0.807	0.773	0.274
locus176	0.293	0.033	0.264	0.371	0.018
locus185	0.516	0.625	0.746	0.196	0.668
locus194	0.628	0.563	0.378	0.106	0.326
locus203	0.512	0.565	0.448	0.511	0.043
locus209	0.464	0.742	0.753	0.662	0.200
locus220	0.576	0.541	0.515	0.469	0.041
locus254	0.149	0.016	0.195	0.020	0.782
locus333	0.013	0.009	0.018	0.009	0.527
locus427	0.693	0.361	0.382	0.297	0.918

### Phenotypic Differences in Height and Floral Colour

Phenotypic variation was present between populations. Floral colour frequencies differed significantly between sites (X^2^ = 30.78, df  = 8, p<0.001; Figure S1), with almost all individuals from the alpine sites having white flowers. Plant height differed significantly between sites (ANOVA, F_4,118_ = 8.83, p<0.0001; Figure S1), as those at HSB-alpine were shorter than plants from other sites (Tukey’s test, p<0.05). No loci had a significant association with floral colour or above ground height (Spearman correlation, df  = 121, p>0.05).

## Discussion

In this study we conducted a genome scan for signatures of natural selection in alpine and lowland populations. We decompose genetic variation across the genome into outlier and non-outlier loci to examine the putative effects of natural selection and selectively neutral evolution to population differentiation. Amongst all alpine and lowland sites an estimated 2.7% of the genome (1.9% with peak height data) represented possible signatures of natural selection, within the 1–4% range reported from other studies of species that occupy contrasting environments [37], [80]–[82]. Frequencies of outlier loci varied independently, suggesting that they were not in linkage disequilibrium. These loci were highly differentiated between populations, as expected from divergent selection, with no evidence for balancing selection at any locus. The particularly high F_ST_ between alpine and lowland sites suggests accelerated genetic differentiation at the outlier loci in alpine environments in the Alberta region.

### Population Divergence at Outlier and Neutral Loci

The existence of population structure at both neutral and outlier loci suggests natural selection and neutral evolution have related effects in alpine and lowland population divergence. Neutral population divergence between alpine sites suggests the processes such as genetic drift, restricted gene flow or other demographic parameters may have affected the evolution of outlier loci in alpine environments in addition to the putative effects of natural selection. However, outlier levels of genetic differentiation at a number of loci, despite the lack of complete population differentiation in some alpine populations, suggests natural selection can affect ecotypic divergence between lowland and alpine populations in the presence of gene flow. The isolated and extreme nature of alpine habitats may generally mean that drift, mutation and other neutral evolutionary processes are necessarily linked to the effects of natural selection in alpine environments, although the degree to which this occurs may depend on levels of gene flow between populations and environments.

Alpine environments generally exert extreme abiotic selection for survival and reproduction, and successful organisms must function at lower temperatures, shorter growing seasons, and exposure to wind, intense radiation, and falling debris [33], [83]. The more temperate abiotic conditions in lowland environments relax selection for extreme abiotic tolerance compared to alpine environments, but biotic stresses such as competition and herbivory may also exert selection [33], [34]. The differences between these environments can eventually cause population divergence through adaptation to extremely different ecological conditions [35]–[37]. Divergent selection has been associated with ecological differences between alpine and lowland environments [37], [38], along elevation, precipitation and temperature gradients [35], [36], [39], [84]–[87], host-use differences [81], [88], [89], and ecological opportunity following major geological events [3], [4], [41]. Our findings are consistent with these studies of divergent natural selection, with the added insight that neutral evolution and selection may be linked processes in alpine-lowland divergence.

Our findings are consistent with other studies in underscoring the importance of considering neutral evolutionary factors when inferring cause of evolution at outlier loci or traits under selection [90]–[93]. Our study contributes by emphasizing the importance of neutral population structure analyses in the context of population genomics, and is an important step in accounting for alternate hypotheses that could explain outlier levels of genetic variation in genome scans for signatures of natural selection [32]. Neutral evolution in some cases is predicted to lead to heightened differentiation characteristic of outlier loci, particularly if certain populations have shared ancestry, undergone recent bottlenecks, or if barriers to gene flow are more prevalent between a subset of populations [94], [95]. Neutral mutations that arise in growing populations can also appear to be highly differentiated, as they increase in frequency as the population expands [96], [97], but are unrelated to adaptation. Additionally, the outlier nature of genetic differentiation at some loci may not be caused by environmentally based natural selection. Loci associated with genetic incompatibilities between populations, which can be heightened by natural selection between divergent environments, may be the primary cause of increased differentiation at the outlier loci [32], [98]. Selection against newly arisen deleterious mutations in a population can cause local differentiation at the mutated locus that would appear similar to other loci associated with adaptation [99].

While both natural selection and neutral evolution appear to have affected population divergence, the relatively small sample of populations limits the implications of these results to the regional level. Further sampling of populations from both lowland and alpine environments across the range of the species will add to these results and aid in the determination of how population divergence between environments and amongst alpine populations has occurred in this species. The utilization of AFLP for genome scans has limitations. AFLP loci may be associated with a particular region of the genome, possibly missing section of the genome that are under natural selection [100]. In addition to the relatively few loci available for the genome scan, this concentration of restriction sites may account for the failure of AFLP to find outliers [101]. The anonymous nature of AFLP markers restricts inference into the potential function of outlier loci, and makes it difficult to determine which evolutionary processes may be acting on a particular locus. The acquisition of sequence information and further experimentation to determine if outlier alleles directly affect fitness will be important for separating the effects of neutral and selective evolution and establishing a mechanistic link between genotype, phenotype and natural selection. Nonetheless, by separately analyzing population structure at both neutral and outlier loci, we are able to determine which populations outlier loci were more likely to have evolved due to environmentally based natural selection (i.e. adaptation to alpine and lowland environments), and which populations may be good candidates for alternate explanations for outlier loci.

### Phenotypic Differences between Alpine and Lowland Populations

The high frequency of white-flowered individuals in alpine environments, and the shorter plants at an alpine site suggests these phenotypes reflect differences between alpine and lowland environmental conditions. Low shoots can reduce damage from exposure to wind and falling debris, and closer proximity to the ground can also limit freezing and frost formation [33], [83]. The high frequency of white flowers in alpine environments could indicate differences in the pollinator community at alpine sites has favoured white floral colour. Alternatively, the lack of pigmentation could be the by-product of lower phytochemical production from generally lower herbivory in alpine environments [33]. The lack of correlation between genotype and phenotype for both floral colour and shoot height traits could indicate that the genome scan included too few loci to detecting such associations, or these traits could be phenotypically plastic, as is often observed [102]. A greater coverage in genome scans, particularly in polyploids that tend to have large genomes, may be required to uncover the genetic basis of these potentially adaptive phenotypes.

### The use of AFLP Peak Height and Dominant Data for Genome Scans

The higher false discovery rate and fewer detected loci based on peak height data than dominant data suggests peak height is more conservative in the identification of outlier loci. This contrasts with another investigation that found band intensity had a higher power to detect outlier loci than did dominant data [37]. Polyploidy may cause ambiguity in genotyping based on peak height. For tetraploids, the potential for up to four alleles at each locus should introduce greater variation in peak height than in diploids, possibly leading to a greater variation in estimates of genetic differentiation and/or decreased ability to detect outlier loci due to homoplasy. If so, band intensity estimates of the number of outliers in polyploid species would be more conservative than in diploid species. Additionally, [37] may have involved different selection intensity, such that estimates of outliers may depend on biological factors other than the simple presence/absence of natural selection. The potentially higher power to detect outlier loci with band intensity data should be tested further, although the utility of this data may be limited to diploid species. The utilization of high-throughput sequencing approaches may ultimately be the best method of circumventing the limitations of conventional AFLP.

### Conclusions

Population divergence is in many cases caused by divergent natural selection in species that span different environments, but can also be linked to or caused primarily by neutral evolution. A detailed analysis of population structure at both neutral and outlier loci as we conducted in this study can elucidate the relative contribution of selection and neutral evolution to population divergence, and is an important component of the determination of the causes of outlier levels of genetic variation. The interaction of selection and neutral evolution may be a prominent mode of population divergence amongst alpine populations. Further sampling of alpine populations in multiple species will determine if this is generally true for plants, while the incorporation of high-throughput sequencing may reveal the identity and putative function of loci associated with alpine and lowland adaptation. An accurate determination of the extent of neutral population differentiation in conjunction with the potential effects of natural selection will remain an important component of inferring the relative contribution of these evolutionary processes to variation across the genome in natural populations.

## Supporting Information

Figure S1 Variation in plant height and flower colour within and among lowland sites, BHS, BL and WC, and alpine sites, HSB and HWP.
(TIFF)
