# Supplementary figures and images for: Natural Selection and Neutral Evolution Jointly Drive Population Divergence between Alpine and Lowland Ecotypes of the Allopolyploid Plant Anemone multifida (Ranunculaceae)
Source: PLoS One. 2013 Jul 18;8(7):e68889. doi: 10.1371/journal.pone.0068889 (PMC3715535; doi:10.1371/journal.pone.0068889)

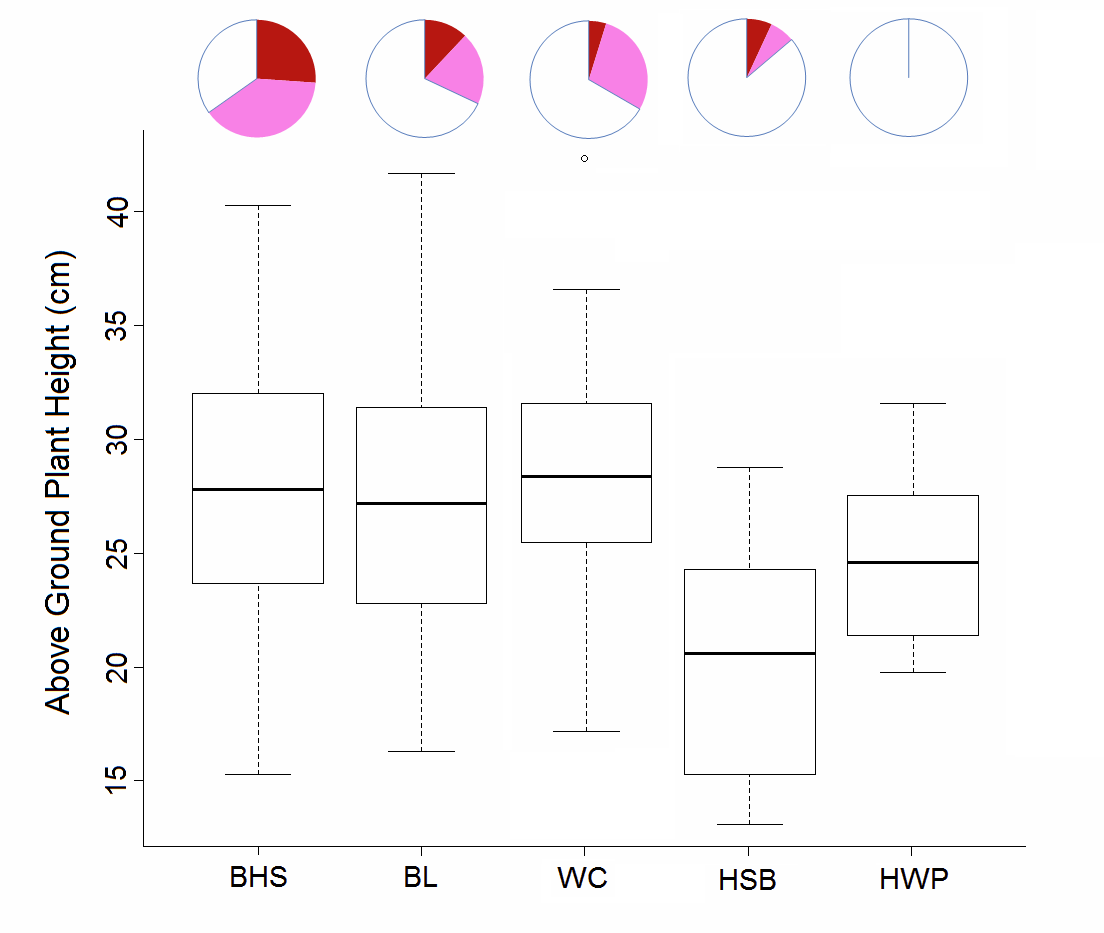

Supplement: Figure S1 — Variation in plant height and flower colour within and among lowland sites, BHS, BL and WC, and alpine sites, HSB and HWP. (TIFF) [file pone.0068889.s001.tiff]
